# A Novel Index Measure of Housing-related Risk as a Predictor of Overdose among Young People Who Inject Drugs and Injection Networks

**DOI:** 10.21203/rs.3.rs-3083889/v1

**Published:** 2023-06-26

**Authors:** Kathleen Kristensen, Leslie D. Williams, Charlie Kaplan, Juliet Pineros, Eunhye Lee, Maggie Kaufmann, Mary-Ellen Mackesy-Amiti, Basmatee Boodram

**Affiliations:** University of Illinois Chicago School of Public Health; University of Illinois Chicago School of Public Health; University of Illinois Chicago School of Public Health; University of Illinois Chicago School of Public Health; University of Illinois Chicago School of Public Health; University of Illinois Chicago School of Public Health; University of Illinois Chicago School of Public Health; University of Illinois Chicago School of Public Health

**Keywords:** housing instability risk, overdose, people who inject drugs, Chicago, negative binomial regression

## Abstract

**Background:**

For people who inject drugs (PWID), housing instability due to decreasing housing affordability and other factors (e.g., loss of housing due to severed relational ties, evictions due to drug use) results in added pressure on an already vulnerable population. Research has shown that housing instability is associated with overdose risk among PWID. However, the construct of housing instability has often been operationalized as a single dimension (e.g., housing type, homelessness, transience). We propose a multi-dimensional measure of housing instability risk and examine its association with drug overdose to promote a more holistic examination of housing status as a predictor of overdose.

**Methods:**

The baseline data from a network-based, longitudinal study of young PWID and their networks living in metropolitan Chicago, Illinois was analyzed to examine the relationship between a housing instability risk index—consisting of five dichotomous variables assessing housing instability—and lifetime overdose count using negative binomial regression.

**Results:**

We found a significant positive association between the housing instability risk score and lifetime overdose count after adjusting for 12 variables.

**Conclusions:**

Our results support the practical utility of a multi-dimensional measure of housing instability risk in predicting overdose and highlight the importance of taking a holistic approach to addressing housing instability when designing interventions.

## BACKGROUND

Opioid overdose has become an increasingly salient public health crisis in the United States in recent years. According to Centers for Disease Control, U.S. overdose deaths increased by 28.5% from Spring of 2021 to Spring of 2022 (1). For people who inject drugs (PWID), the overdose crisis is compounded by the co-occurring affordable housing crisis in the U.S., particularly in major cities, where stable housing has become increasingly difficult to obtain. Between 2021 and 2022 home rental and apartment rental pricing increased by over 10% and 20%, respectively in major metropolitan areas (2), which has fostered an increase in homeless populations with the latest data showing a 2% increase in homelessness from 2019 to 2020 (3).

A growing body of research has highlighted the importance of addressing structural factors, including housing, that contribute to substance misuse and its adverse outcomes such as overdose (4–10). PWID are particularly susceptible to recurrent housing instability (i.e., lack of access to stable housing) due to ongoing barriers to maintaining and accessing housing (11, 12). As a result, PWID who begin to experience housing instability often remain in a state of long-term, cyclical homelessness (12). Theoretically, housing instability can increase stress, depression, and anxiety, all of which are associated with increased risk for overdose (13–21). Housing instability is also directly associated with increased risk of overdose in some (5, 9, 14, 22–27), but not all studies (28–30), perhaps partly due to the inconsistent conceptualizations of housing instability across studies. Mechanisms that might explain this relationship include more intense or risky patterns of substance misuse among homeless PWID (31–33), and rushed injection in public spaces (34–36). Recent incarceration entailing loss of tolerance may also play a role (37, 38).

Most prior overdose-focused research studies have measured housing stability using three main constructs—unstable residence, homelessness, and residential mobility/transience—operationalized in varying ways. Multiple studies have used a dichotomous measure that identifies housing as stable or unstable using the type of housing (e.g., home, shelter) that individuals have recently resided in to make this determination as the indicator (4–6). Alternatively, a dichotomized measure of homelessness has been used by researchers, but this categorization is inconsistently determined by either self-report or using individuals’ recent housing type (22, 24, 26, 27). Studies have also examined housing status using the construct of residential mobility/transience, which is typically measured using the number of residences or communities lived in during a period of time (8, 9, 14, 28, 39).

Research examining the relationship between housing and opioid overdose typically has focused on a single component of housing instability, the most common of which are housing type, housing tenure, or self-reported homelessness. Housing type is typically based on the type of residence (e.g., house/apartment, hotel/motel, abandoned building, shelter, etc.) (4, 5, 23, 24, 26, 29, 30); housing tenure dichotomizes stable and unstable housing or residential mobility/transience based on the length of time an individual has lived in a location or alternatively based on the number of moves an individual has undergone in a defined time period (14, 16, 19, 39); and homelessness relies on self-reported homelessness or determinations based on housing type to identify individuals as homeless (22, 24, 25). Used alone, each operationalization is limited in capturing housing instability. For example, for housing type, what constitutes stable residence varies in the literature and often does not account for length of residency at each type (8, 9, 14, 28, 39–41). Moreover, housing tenure does not capture the quality of the residence, and therefore, may incorrectly identify long-term stays in unsafe environments as stable housing (42). Finally, self-reported homelessness is often defined by varying lengths of time and severity (e.g., live in a shelter or street) or unclearly defined, which could lead to biased responses due to varying definitions of homelessness (41).

To address the limitations of single-factor measures of housing instability, Frederick et al. proposed that housing instability measures should be multi-dimensional, an argument that is supported by other housing researchers attempting to improve measures of housing instability (41, 43, 44). Specifically, Frederick et al. suggested a measure that includes assessments of housing type, tenure, legal status (criminal justice involvement), employment, and income (41). Frederick et al. argued for the inclusion of items that affect stability such as employment and legal status to measure risk of housing instability over time, rather than a static condition of housing (41). Dynamic measures of housing instability such as this have been called for to address the inherently fluid nature of housing status (45, 46).

To some extent, researchers have heeded the call for more multi-dimensional measures of housing stability by combining constructs or increasing the number of categories within constructs in an effort to create more robust measures of housing instability. Examples include (i) Whittaker et al. (2015), who used housing type to dichotomously categorize individuals as homeless or stably housed; (ii) Calvo et al. (2017) who combined homelessness and stable housing using a three-category measure; and (iii) Perez-Figueroa et al. (2021), who created a four-category (unhoused, imminent risk of homelessness, precarious housing, and stable housing) measure of housing stability based on housing type. These studies go beyond the commonly used dichotomous measures of housing instability; however, they do not go so far as to account for factors that can impact housing instability risk such as employment, income, and criminal justice involvement as called for by housing researchers, and they still only address one dimension of housing instability (41, 43, 44). Therefore, there is a need to unify these constructs under a single index measure of housing instability risk in order to better understand the role of “housing status” more holistically as a potential predictor of overdose and other substance use-related outcomes.

Our study will bridge the gap between opioid overdose and housing literature by examining the relationship between a multi-dimensional measure of housing instability and lifetime overdose count among a cohort of young PWID in the Chicago Metropolitan Area, which includes the city of Chicago (Cook County) and the surrounding suburbs in Cook County and its adjacent counties that are also the five most populous counties after Cook County (DuPage, Kane, Lake, McHenry, and Will Counties). To holistically encapsulate housing instability risk, the measure will consider housing tenure, housing type, and self-reported homelessness as well as employment, income, and criminal justice involvement (i.e., prior incarcerations). Unifying these factors into a single measure of housing instability risk is proposed to reduce inconsistency in the constructs used to capture housing instability when examining its relationship with overdose.

## METHODS

### Sample and Recruitment

We used baseline data from on ongoing (October 2018-March 2020) longitudinal study examining young (18–30 years old) PWID and their egocentric (personal) network members (of all ages) – including the members of their injection, sexual, and social support networks (i.e., anyone the participant had injected with, had sex with, or received money from in the past six months, respectively). Detailed study methods have previously been described (47). In brief, to be eligible, primary participants (i.e., egos—those directly recruited by staff into the study) had to (i) be between 18–30 years old, (ii) have injected drugs at least once in the past month, (iii) be able to speak English, and (iv) be a resident of the city of Chicago or surrounding suburbs during the past 12 months. Members of the egos’ injection networks (i.e., alters—those directly recruited by egos) were eligible for enrollment if they were (i) at least 18 years old, (ii) able to speak English, (iii) a resident of the city of Chicago or surrounding suburbs during the past 12 months, and (iv) referred to the study by an ego. Age was verified using a driver’s license or state-issued ID. When participants did not have an ID, study staff assisted with obtaining one. To verify participants’ injection status, injection stigmata (i.e., physical signs of injection on the skin) were inspected by study staff; when signs of injection were not evident, a standardized list of questions from earlier studies was administered to evaluate participants’ knowledge of the injection process.

Recruitment was primarily conducted at two field sites of Community Outreach Intervention Projects (COIP), a center established in 1986 within University of Illinois at Chicago School of Public Health that currently (i) provides services (e.g., syringe service programs - SSPs, naloxone distribution, case management, and medication assisted treatment - MAT - services) and (ii) conducts research with PWID to better inform intervention efforts. These field sites are located in Chicago areas with higher-than-average rates of HIV, hepatitis C, sexually transmitted infections, and drug-related arrests (21). To diversify the pool of participants and target non-SSP-involved and suburban PWID, recruitment was also done directly in open outdoor drug market areas known from previous studies by our group to attract both urban and suburban PWID and though mobile unit outreach postings at community-based organizations serving PWID, as well as through social media platforms (48).

At the baseline visit, egos were asked to recruit up to five alters, defined as people with whom they used drugs in the past six months in the same space. To recruit these alters, egos were given up to five coupons linked to the recruiting ego via an alphanumeric code. Coupons were only redeemable by alters named by an ego in their baseline survey, and identities were verified using a driver’s license or state ID.

### Data Collection

Data collection was primarily conducted at the two COIP field sites and supplemented using a mobile van. However, with the advent of the COVID-19 pandemic (March 2020), data collection was shifted to virtual via the Zoom videoconferencing platform to protect the safety of participants and study staff. All ego and alter participants completed a process of informed consent prior to data collection. All study procedures were approved by an Institutional Review Board at the University of Illinois at Chicago. Because the time needed to complete data on participants’ networks can vary greatly based on network size, participants were compensated hourly for their participation in the study. Specifically, they were compensated at a rate of $20/hour. The average interview length was 2.5 hours, thus resulting in an average compensation amount of $50 per interview. Additionally, ego participants received $20 for each alter recruited to the study.

All COIP services (SSP, HCV/HIV testing, counseling, case management, linkage to care, etc.) were made available to all participants and to anyone recruited to find out more about the study, regardless of study enrollment status.

### Measures

#### Outcome: Lifetime Overdose Count

The outcome of interest is the number of self-reported lifetime overdose. Overdose was defined to participants as having passed out, turned blue, or stopped breathing from using drugs. Participants were asked about the number of times they had ever overdosed on opioids, other hard drugs, or painkillers in their lifetime.

#### Primary Covariate: Housing Instability Risk Index

The housing instability risk index was the primary independent variable and consisted of the sum of five dichotomous variables—each representing a different domain of housing instability as presented in theory by Frederick et al. —resulting in index scores from 0–5 (41). Measures of (i) housing type and tenure, (ii) self-assessed homelessness, (iii) monthly income, (iv) employment, and (v) criminal justice involvement were dichotomized into responses that did and did not indicate “instability.” Cutoff points for this dichotomization process were selected based on theoretical and empirical rationales as described below. The descriptive statistics for each of the variables making up the housing risk instability score are included in the online supplement (Table S1).

For the *housing type and tenure domain*, individuals who met the definition of unstable on either criterion were deemed unstable. Housing type, as designated in the literature, is deemed unstable if places lived in the past six months include vehicles, public transportation, abandoned buildings, shelters or welfare residences, jail, prison, a detention center, juvenile hall, or on the streets. For the tenure criterion of this domain, participants who reported lived in three or more different places in the previous year were categorized as unstable (4, 23, 24, 29, 30, 40). This definition represents a more severe level of transience than in similar studies of PWID that used two or more as the cut-off, including one by our team (49, 50). For the *homelessness domain*, instability was deemed if the participant answered yes to the survey question: Have you ever been homeless in the past six months? For the, *monthly income domain*, instability was indicated if an individual reported an income of less than $999 per month in the past six months, a cutoff point that was selected based on the distribution of income amounts reported by our sample (specifically, less than $999 per month was the lowest quartile of reported income) and given that this amount is below the poverty threshold for a single individual (51). For the *criminal justice involvement domain*, instability was assessed by the following survey question: How many times have you been to jail in your lifetime? Number of times in jail was used rather than ever in jail because the majority of our sample of PWID had been to jail at least once. Based on our sample’s distribution, going to jail eight or more times (ever) was determined to be indicative of instability as this number was the cutoff for the upper quartile. For the *employment domain*, instability indicated primary source of income in the past six months was from temporary work, panhandling, recycling, state/federal benefits, public assistance, family/friends, theft, selling drugs, or sex for money as these categories did not indicate stable, legal employment or self-employment as the main source of income. Although non-traditional or illicit sources of income are not all inherently unstable, since individuals can sometimes reliably support themselves with these incomes, these types of employment can present challenges in obtaining housing as they affect one’s ability to successfully apply for housing (52).

#### Other Covariates

Demographic characteristics of race/ethnicity (non-Hispanic white, non-Hispanic Black, Hispanic, and Mixed/Other), post high school education (completed post high school education or not), gender (male, female, or transgender [all]), and age (number of years) were examined as covariates given evidence from extant literature supporting a relationship with overdose, and/or due to interest in adjusted effects after controlling for these demographic characteristics (53–55). Other covariates listed below were examined based on theoretical and empirical support of their potential confounding of the association between housing instability and lifetime overdose count. Backloading (i.e., mixing drugs in a single syringe and then squirting into others’ syringes) and syringe sharing were included as potential covariates given known associations between injection risk behaviors and overdose and known associations between housing instability and increases in injection risk behaviors (18, 56, 57). Participants were asked how often they engaged in backloading and syringe sharing behaviors in the past six months with five response options: never, less than half the time, about half the time, more than half the time, or always. Given that network characteristics have been shown to predict overdose among PWID and network characteristics can theoretically be impacted by housing status, we included mean ego-alter tie strength (which is an indicator of relationship strength) and core network size as potential covariates (58–62). Depression and stigma, measured using the Center for Epidemiologic Studies Depression Scale (CESD-10) and the Substance Use Stigma Mechanisms Scale (SU-SMS), respectively, were also included as covariates, as high levels of depression and stigma are known to be associated with increased overdose risk (20, 63), and as housing instability has been shown to be related to depression and stigma (64, 65). Public versus private injection spaces were also included as potential covariates, as housing instability can impact one’s access to private injection spaces and public injection has been shown to be associated with increased overdose risk (66). Participants were asked to list all injection locations in the past year and to characterize the type of place (specific responses options included: my house, friend/relative’s house, car, public place, public transportation, abandoned building, hotel, motel, SRO, on the street, other) for each injection location. The place types of “my house” and “friend/relative’s house” were considered private and all other place types were considered public. If a participant injected in only public places or in only private places in the past year, they were coded as a public injector only or as a private injector only, and if a participant injected in both public and private places in the past year they were coded as both a public and private injector. Residential categories (urban, suburban, transient) were also included as potential covariates due to known relationships between housing instability and urban areas, as well as to extant literature suggesting a relationship between urban or non-urban residence and overdose (67). Participants’ residence location was based on all listed residences for the past year and was categorized as urban (Chicago residences only), suburban (non-Chicago residences only), or transient (both Chicago and non-Chicago residences.

### Statistical Analysis

Out of the 334 participants for whom baseline data were collected, 17 were missing data for analytic variables and were not included in the final analysis (N = 317). Proportions of missingness for each variable are presented in the online supplement (Table S2). All analysis was completed in SAS v. 9.4. First, a descriptive analysis was completed for all analytic variables. Bivariate Pearson correlations were computed for all independent variables to check for multicollinearity. Negative binomial regression was deemed most appropriate since the outcome of interest, lifetime overdose, is a count variable and Poisson regression was infeasible due to overdispersion in our sample (mean = 5.10 variance = 49.65). Bivariate negative binomial regression models were conducted that regressed lifetime overdoses on the main predictor (the housing instability risk index) or on one of the potential covariates (see Measures above). An adjusted negative binomial regression model was then generated that included the housing instability risk index, covariates that were found to be significantly related to (p > .05) lifetime overdoses in our bivariate analyses, and some additional demographic covariates that were included despite no significant findings, due to strong support from the extant literature for their importance as covariates or potential confounds.

## RESULTS

### Descriptive and Bivariate Analysis

Table 1 reports descriptive statistics on key covariates, including the primary predictor (housing instability index), injection behaviors, network characteristics, and the outcome (lifetime overdoses) for the full sample (n = 334). The sample was mostly non-Hispanic/Latinx white (59.9%), male (72.5%), with mean number of lifetime overdoses of 5.10 (standard deviation [s.d.] = 7.05) and the mean housing instability risk score was 2.95 (s.d.=1.29) (Table 1).

Bivariate correlations between all independent variables suggested that multicollinearity was not a concern for the present set of independent variables. Table 2 summarizes the negative binomial regression models that tested bivariate associations between variables that have been shown to be theoretically related to housing instability and overdose. The housing instability risk score in addition to age, stigma, depression, public injection, private injection, backloading, and syringe sharing were significantly associated with lifetime overdose count, while residence location (e.g., urban or suburban) and network characteristics were not significant and therefore were excluded from the adjusted negative binomial regression model (Table 2).

### Final Negative Binomial Regression Model

The final negative binomial regression model (see Table 3) used the analytic sample (N = 317) and included as independent variables the housing instability index and 12 covariates of interest. Goodness of fit statistics indicated good model fit (log likelihood= −826.57; AIC = 1697.14; and BIC = 1779.83). Two constructs were significantly associated with lifetime overdose count: housing instability risk score (Wald Chi-Square = 18.35, p = .003) and depression (Wald Chi-Square = 12.55, p < .001). Higher values of these two variables were associated with a greater number of lifetime overdoses as indicated by incident rate ratios (IRR) displayed in Table 3. Table 3 shows that for housing instability scores from 1–5, as the housing instability risk score increases, the incidence rate of overdose increases. However, for a housing instability risk score of 0, the IRR (IRR = 0.37) was higher than that of a housing instability risk score of 1 (IRR = 0.32) or 2 (IRR = 0.36), thereby deviating from the trend of overdose IRR increasing as housing instability score increases. One potential explanation for this deviation from the positive relationship observed for all other housing instability scores is the higher standard error in the IRR estimation due to only 11 individuals in the sample having a housing instability risk score of 0.

## DISCUSSION

Our study examined a novel measure of housing instability, conceptualized as a combination of housing type, housing tenure, employment, monthly income, and criminal justice involvement as a predictor of overdose risk among young people who inject drugs and their injection networks from a large metropolitan area that includes both urban and suburban residents. We found a strong positive association between this housing instability risk score and lifetime overdose count. Additionally, depression was associated with increased lifetime overdose count. The attenuation of associations between overdose and age, syringe sharing, backloading, private injection, and public injection in the full model suggests that these associations are likely to a large degree due to confounding with housing instability risk and/or depression. To our knowledge, this study is the first to examine overdose occurrence as it relates to a holistic, multi-dimensional measure of housing instability risk. The significant findings indicate the usefulness of such a measure in examining housing instability risk as a risk factor for overdose.

As critics of one-dimensional measures of housing instability argue, housing instability is not a static condition that can simply be captured by an individual’s current residential status (41, 43, 45, 46). For example, one’s financial situation or criminal justice involvement can quickly destabilize a previously stable housing situation. Characterizing housing status as housing instability risk overcomes part of this problem by considering predictive factors of stability that can indicate whether a person is at risk to become unstably housed. Conceptualizing housing status as a continuum of instability risk is essential to inform early intervention for PWID, as once they become unstably housed, continually increasing barriers may inhibit return to housing stability. However, predictive factors of stability cannot fully capture the degree to which an individual’s housing status may be changing over time. Using housing pathways that granularly track housing changes day-to-day has been suggested by some researchers, but collecting that data is arduous and time-consuming, and for the present study goes beyond the scope of available data (45, 46). Roy et al. conducted such a study examining high-risk HIV behaviors as an outcome of residential trajectory measured three months at a time exemplifying such methodology is possible, but this granular housing data is not obtainable in many contexts (68).

Given the association found between the housing instability risk index and the number of lifetime overdoses, intervention efforts aimed at addressing housing for PWID should focus on a holistic definition of housing instability risk and consider factors beyond an individual’s current residence that may be impacting their housing status. Additionally, future research on PWID should consider the use of this holistic approach to characterize housing instability risk to consistently capture a wider range of factors impacting housing instability and to avoid confusion in the literature that results from inconsistent use of constructs to represent housing instability.

### Limitations

The current study is limited by the cross-sectional study design, i.e., given that the current study only used baseline data, a temporal relationship between housing instability risk and overdose cannot be established. For PWID, current housing instability risk is often the result of a process of destabilization that has taken place over years, and the limitations of cross-sectional data does not allow for empirical explication of this process. Instead, a measure of recent housing instability risk had to be used to capture the extent to which participants have been impacted by this destabilization process overtime as reflected by their current housing instability risk. In addition, cross-sectional data cannot capture how included covariates (e.g., syringe sharing, backloading, depression) might change over time in response to experiences of housing instability or overdose. Several of the measures making up the housing instability risk score in this study were also limited in their operationalization due to available data. Criminal justice involvement was measured using data on the lifetime number of times jailed, which is limited to one specific indicator of criminal justice involvement as the available data on prison was highly homogenous. Additionally, income was measured as a categorical variable, limiting the data to the cutoff points established by the survey question. As a result, the unstable income category included a wider range of incomes than would be ideal. Future research should include a more robust measure of criminal justice system involvement and more granular assessment of income level. The self-reported nature of these measures also presents a limitation as many of the responses relied on individuals recalling information from the past; however, the time frame for most questions was the last 6 months. Lastly, this study is limited in its generalizability as it only examines young PWID from Chicago and the surrounding suburbs. Future research should expand on the application of a holistic measure of housing instability risk by examining its usefulness in studying other populations of PWID.

## CONCLUSIONS

Through the examination of the relationship between a holistic index measure of housing instability risk and the number of lifetime overdoses, the present study addresses the need to use multi-dimensional measures of housing instability when examining the association between housing and overdose. The significant association found in this study between a five-dimension housing instability risk score and lifetime overdoses supports the utility of such measures. While future research is needed to examine applications of multi-dimensional measures of housing instability risk in contexts outside of that of the present study, the present findings support the need to move beyond one-dimensional understandings of housing both in research settings and when designing interventions.

## Figures and Tables

**Table 1:** Study population characteristics (N = 334)

Variable				N (Valid %) or Mean (SD)	
Male				242 (72.5%)	
Female				91 (27.3%)	
Transgender				1 (0.3%)	
Hispanic				83 (24.9%)	
Non-Hispanic White				200 (59.9%)	
Non-Hispanic Black				31 (9.3%)	
Mixed/Other				20 (6.0%)	
Post High School Education				128 (38.3%)	
Age				30.53 (7.69)	
Lifetime Overdoses				5.10 (7.05)	
Housing Instability Risk Score				2.95(1.29)	
Depression				2.04 (0.48)	
Stigma				2.99 (0.51)	
Public Injection only				135 (41.3%)	
Private Injection only				74 (22.6%)	
Both Private and Public Injection				118 (36.1%)	
Chicago Resident Only				119 (36.2%)	
Non-Chicago Resident Only				110 (30.4%)	
Chicago and Non-Chicago Resident				110 (33.4%)	
Core Network Size				5.65 (3.01)	
Mean Ego-Alter Tie Strength				3.13 (0.55)	
	Never	<Half the Time	~Half the Time	>Half the Time	Always
Backloading	203 (62.7%)	97 (29.9%)	13 (4.0%)	7 (2.2%)	4 (1.2%)
Syringe Sharing	186 (57.4%)	105 (32.4%)	23 (7.1%)	7 (2.2%)	3 (0.9%)

**Table 2:** Bivariate Negative Binomial Regression of Predictors on Lifetime Overdose (N = 334)

Variable	Wald Chi-Square	p-value
Male		
Race/ethnicity		
Non-Hispanic White	2.09	.149
Non-Hispanic Black	1.25	.263
Hispanic	0.08	.780
Post High School Education	0.18	.675
**Age**	**4.57**	**.033**
**Housing Instability Risk Score**	**28.78**	**<.001**
**Depression**	**21.99**	**<.001**
**Stigma**	**9.61**	**.002**
**Public Injection only**	**8.74**	**.003**
**Private Injection only**	**12.71**	**<.001**
Chicago Resident Only	.026	.872
Non-Chicago Resident Only	2.82	.093
Network Core Size	2.40	.121
Mean ego-alter tie strength	2.90	.088
**Backloading**	**18.42**	**.001**
**Syringe Sharing**	**14.29**	**.006**

**Table 3:** Negative Binomial Regression Model Predicting Lifetime Overdose (N = 317)

Variable^a^	β^b^	Std. Error of β	Wald Conf. Interval of β		IRR^c^
Housing Instability Risk Score			Lower	Upper	
**0**	**−0.99** *	**0.43**	**−1.83**	**−0.15**	**0.37**
**1**	**−1.14** ***	**0.31**	**−1.76**	**−0.53**	**0.32**
**2**	**−1.02** ***	**0.27**	**−1.55**	**−0.50**	**0.36**
**3**	**−0.83** ***	**0.25**	**−1.33**	**−0.34**	**0.43**
**4**	**−0.64** **	**0.23**	**−1.10**	**−0.18**	**0.53**
**Depression**	**0.51** ***	**0.15**	**0.23**	**0.80**	**1.67**
Stigma	0.19	0.13	−0.07	0.45	1.21
Backloading					
1	−0.60	0.61	−1.80	0.61	0.55
2	−0.18	0.60	−1.36	0.99	0.83
3	−0.45	0.64	−1.70	0.80	0.64
4	−0.58	0.73	−2.02	0.85	0.56
Syringe Sharing					
1	−0.49	0.68	−1.82	0.84	0.61
2	−0.38	0.69	−1.74	0.97	0.68
3	−0.16	0.74	−1.61	1.29	0.85
4	−0.23	0.83	−1.85	1.39	0.80
Public Injection = 0	−0.15	0.16	−0.47	0.16	0.86
Private Injection = 0	−0.10	0.19	−0.47	0.27	0.90
Age	−0.02	0.01	−0.04	0.00	0.98
Male = 0	0.01	0.16	−0.31	0.32	1.01
Post High School Ed. = 0	−0.16	0.14	−0.43	0.12	0.85
Dispersion Parameter	1.04	0.11	0.84	1.27	

a Reference Categories: Housing Instability Risk Score = 5, Backloading = 5, Syring Sharing = 5

Public Injection = 1, Private Injection = 1, Male= 1, Post High School Ed.=1

b *p < 0.05, **p < 0.01, ***p < 0.001

c IRR: incidence rate ratios.

## Abbreviations

PWID: People who use drugs
COIP: Community Outreach Intervention Project
SSP: Syringe service program
MAT: Medication assisted treatment
HIV: Human immunodeficiency virus
HCV: Hepatitis C Virus
CESD-10: Center of Epidemiologic Studies Depression Scale
SU-SMS: Substance Use Stigma Mechanisms Scale
SRO: Single-room occupancy
S.d.: Standard deviation
IRR: Incidence rate ratio
AIC: Akaike information criterion
BIC: Bayesian information criterion
